# Beyond polysomnography: toward an etiology-driven management of obstructive sleep apnea

**DOI:** 10.3389/fneur.2026.1750032

**Published:** 2026-02-24

**Authors:** Min Yin, Ting Wu

**Affiliations:** 1 Department of Otorhinolaryngology, The First Affiliated Hospital with Nanjing Medical University, Nanjing, China; 2 Department of Neurology, The First Affiliated Hospital with Nanjing Medical University, Nanjing, China

**Keywords:** etiology-driven management, multidisciplinary collaboration, obstructive sleep apnea, personalized medicine, polysomnography, treatment phenotyping, upper airway evaluation

## 1 Rethinking the logic of OSA management: from ‘outcome-oriented' to ‘etiology-driven'

Obstructive Sleep Apnea (OSA) is a classic clinical syndrome: it is both the common result of multiple etiologies (e.g., upper airway anatomical narrowing, sleep-related neuromuscular control dysfunction, obesity) and the common source of various systemic complications such as hypertension and cardio-cerebrovascular diseases (1). This “syndrome” attribute inherently necessitates a multidisciplinary perspective and collaboration in its diagnosis and treatment (2).

A fundamental flaw exists in the current mainstream management logic: treatment plans (e.g., Continuous Positive Airway Pressure, CPAP) are often directly derived from the precise assessment of the disease's outcome (Polysomnography, PSG), while systematically bypassing the investigation of the specific etiologies located in the upper airway that cause that outcome. This is analogous to diagnosing cardiac arrhythmia via electrocardiogram (ECG) without further investigating whether its root cause is coronary artery disease, cardiomyopathy, or electrolyte disturbances. While efficient, this “outcome-oriented” model can induce a “therapeutic inertia”: simplifying complex etiological heterogeneity into a single severity metric (e.g., the Apnea-Hypopnea Index, AHI) and consequently narrowing diverse treatment options into a severity-based “default pathway” (1).

This compels us to reflect: can we achieve truly personalized treatment when OSA management decisions fail to address the “root cause of upper airway obstruction”? We argue that the assessment of the obstructive outcome (via PSG) and the assessment of the obstructive cause (via upper airway evaluation) are equally important. Moreover, the evaluation of upper airway obstruction sites should be a preemptive, core diagnostic component. We call for establishing a new management paradigm centered on “etiologic assessment”, aiming to provide a more precise and scientific foundation for shared decision-making for each patient through multidisciplinary collaboration (2).

## 2 Repositioning PSG: from the ‘sole arbiter' to a ‘pillar of physiological assessment'

The diagnosis of OSA should be based on PSG. As the “gold standard”, PSG provides indispensable objective data for confirming the disease, quantifying its severity (e.g., AHI, oxygen saturation), assessing systemic risks (e.g., arrhythmias), and identifying certain physiological endotypes (e.g., REM-related OSA). It serves as a crucial premise for treatment decisions (3).

However, OSA treatment decisions cannot rely on PSG alone. PSG is essentially a “physiological event monitor” and a “severity scorer”, not an “etiology diagnostic tool”. It accurately describes “*how many and how severe”* the apneic events are (the outcome) but cannot answer “*where and why”* they occur (the root cause) (4). Different upper airway obstruction patterns can yield similar AHI values, yet their corresponding optimal treatment strategies may differ drastically. PSG data alone cannot inform these choices.

We propose that PSG, as the “basis for severity stratification and risk assessment”, should be integrated with systematic anatomical evaluation serving as the “locator of upper airway obstructive etiology”. Together, they form the complete diagnostic foundation for personalized therapy, with the former answering “*how severe is the disease”* and the latter answering “*where is the disease”* (5).

## 3 The core value and substance of upper airway obstruction site assessment

The direct cause of OSA is the obstruction of the upper airway during sleep, and the ultimate goal of all treatments (whether surgical or CPAP) is to relieve this obstruction. Therefore, the core value of upper airway obstruction site assessment lies in its role as the nexus connecting etiology and treatment, providing an objective basis for “phenotype-specific therapy”(6). A comprehensive assessment should systematically address the following key questions: the location (level) of obstruction, its pattern (configuration), degree (grade), underlying etiology (structural/functional), consequent physiological impact, and potential corresponding relieving strategies. This shifts therapeutic decision-making from severity-based “trial-and-error” to etiology-informed “prediction”.

The implementation of this assessment should follow a tiered, stepwise approach. Tier 1 (Basic Assessment) includes standardized clinical scoring, physical examination, and office-based awake endoscopy (e.g., with Müller's maneuver), offering a rapid, accessible preliminary judgment of the obstruction level (7). Tier 2 (Advanced Assessment), employed when necessary, may involve Drug-Induced Sleep Endoscopy (DISE) to observe dynamic collapse patterns or imaging studies to quantify craniofacial structure and soft tissue volume (8, 9). These tiers are complementary. Promoting the standardization and widespread adoption of basic assessments like office endoscopy is a key step in elevating overall diagnostic standards.

## 4 Constructing a tiered decision-making framework centered on obstruction site assessment

**Figure 1:**
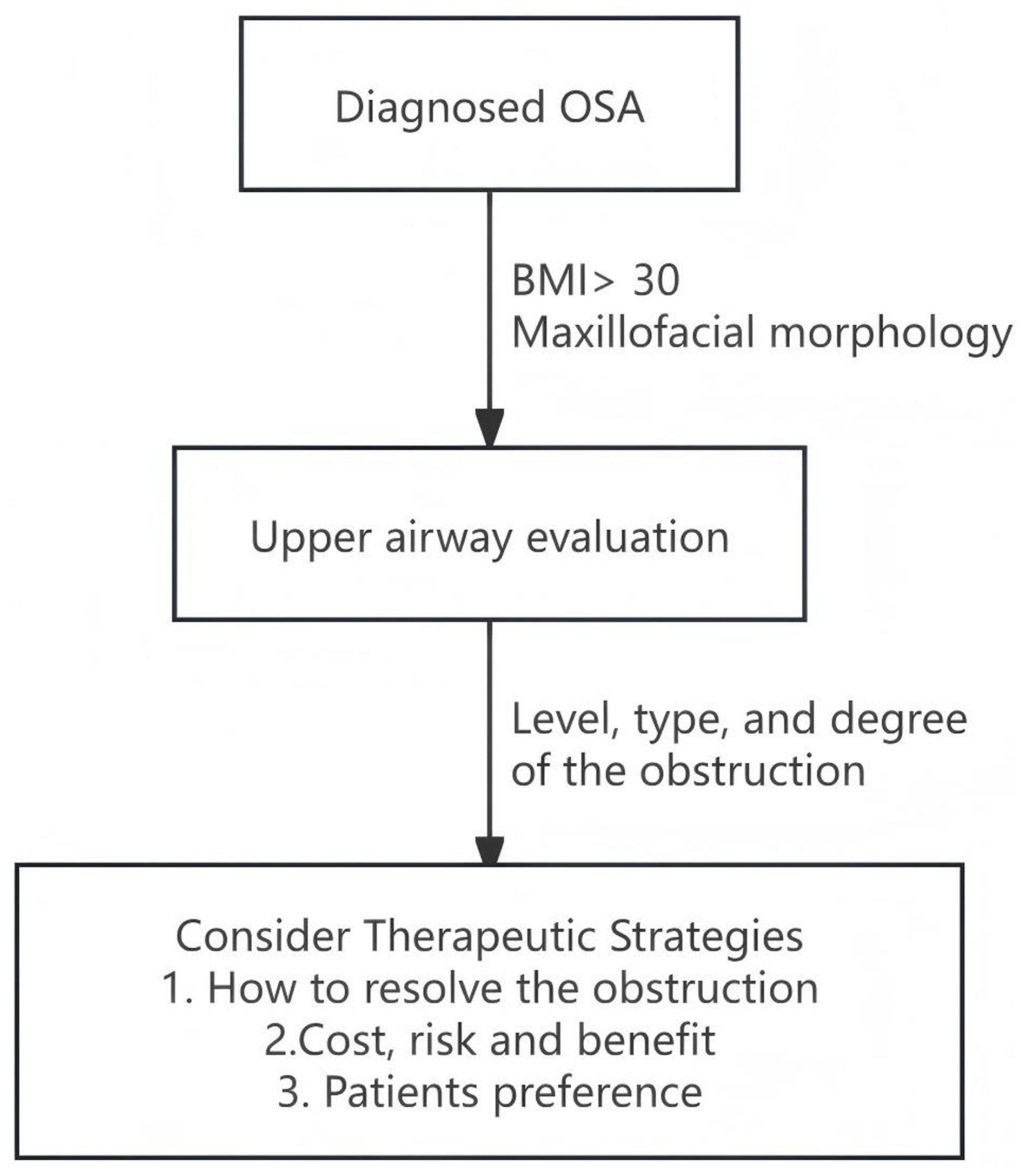
An etiology-driven, four-step clinical decision-making pathway for the management of obstructive sleep apnea.

Based on the above principles, we propose a four-step clinical decision-making pathway centered on obstruction site assessment (Figure 1):

Step 1: Synchronous Initiation of Dual Assessment. Concurrently obtain: (A) Physiological Outcome Assessment: confirm diagnosis and quantify risk via PSG/Home Sleep Apnea Testing. (B) Anatomical Etiology Assessment: identify key etiological information—location, pattern, etc.—of the obstruction via upper airway evaluation, starting with Tier 1 methods.

Step 2: Information Integration and Comprehensive Phenotyping. Synthesize the above data to perform integrated phenotyping (e.g., palatal-dominant, tongue-base-dominant, multilevel, skeletal phenotype), considering the patient's overall context (comorbidities, preferences).

Step 3: Phenotype-Based Collaborative Decision-Making. Formulate an initial management plan within a multidisciplinary framework according to the phenotype: palatal-dominant cases may be assessed for palatal surgery; tongue-base-dominant cases may consider hypoglossal nerve stimulation; skeletal phenotypes may primarily opt for oral appliance therapy; CPAP remains the cornerstone for complex multilevel obstruction, severe OSA, or patient preference. All patients should be counseled on behavioral interventions (10, 11).

Step 4: Implementation, Follow-up, and Re-assessment. Execute the plan with structured follow-up, creating a management feedback loop.

The efficacy of this pathway rests on three core principles:

1. Dual-Source Diagnostic Principle: diagnosis should stem from both PSG (physiological outcome) and upper airway assessment (anatomical etiology) (5).
2. Information Parity Principle: complete information regarding both disease “severity” and “root cause” should be shared between clinician and patient to enable informed decision-making.
3. Integrated Collaboration Principle: the pathway's success depends on institutionalized, protocol-driven collaboration among specialties (e.g., Otolaryngology, Sleep Medicine, Dental Sleep Medicine) based on shared phenotypic information (2).

## 5 Conclusion and future directions: toward an etiology-centric, multidisciplinary era

In summary, polysomnography is the ‘cartographer' mapping the terrain of OSA, while upper airway etiological diagnosis is the ‘spotlight' illuminating the path to treatment. We advocate for a new management framework for OSA syndrome, centered on “etiologic assessment”. This requires:

1. Updating clinical guidelines to grant upper airway anatomical evaluation diagnostic status equal to PSG.
2. Reforming clinical pathways to transition etiologic assessment from an optional adjunct to a standard component.
3. Strengthening multidisciplinary collaboration to achieve integrated decision-making based on shared “anatomical-physiological” phenotypic information (5).

Looking ahead, with advancements in assessment technology and standardization, etiology-driven management will become increasingly precise and accessible. Only by completing this cognitive leap from “phenomenon” to “root cause” can we genuinely tailor the most rational management plan for each individual with OSA, guiding its care into the era of precision medicine.
